# Inhibition of DREAM-ATF6 interaction delays onset of cognition deficit in a mouse model of Huntington’s disease

**DOI:** 10.1186/s13041-018-0359-6

**Published:** 2018-03-09

**Authors:** Alejandro López-Hurtado, Daniel F. Burgos, Paz González, Xose M. Dopazo, Valentina González, Alberto Rábano, Britt Mellström, Jose R. Naranjo

**Affiliations:** 1 Spanish Network for Biomedical Research in Neurodegenerative Diseases (CIBERNED), Instituto de Salud Carlos III, Madrid, Spain; 2 Centro Nacional de Biotecnología, CNB-CSIC, Darwin 3, E-28049 Madrid, Spain; 3 Fundación CIEN, Instituto de Salud Carlos III, Madrid, Spain

**Keywords:** Repaglinide, Neuroprotection, Calcium, NCS-1, Hippocalcin, VILIP

## Abstract

The transcriptional repressor DREAM (downstream regulatory element antagonist modulator) is a multifunctional neuronal calcium sensor (NCS) that controls Ca^2+^ and protein homeostasis through gene regulation and protein-protein interactions. Downregulation of DREAM is part of an endogenous neuroprotective mechanism that improves ATF6 (activating transcription factor 6) processing, neuronal survival in the striatum, and motor coordination in R6/2 mice, a model of Huntington’s disease (HD). Whether modulation of DREAM activity can also ameliorate cognition deficits in HD mice has not been studied. Moreover, it is not known whether DREAM downregulation in HD is unique, or also occurs for other NCS family members. Using the novel object recognition test, we show that chronic administration of the DREAM-binding molecule repaglinide, or induced DREAM haplodeficiency delays onset of cognitive impairment in R6/1 mice, another HD model. The mechanism involves a notable rise in the levels of transcriptionally active ATF6 protein in the hippocampus after repaglinide administration. In addition, we show that reduction in DREAM protein in the hippocampus of HD patients was not accompanied by downregulation of other NCS family members. Our results indicate that DREAM inhibition markedly improves ATF6 processing in the hippocampus and that it might contribute to a delay in memory decline in HD mice. The mechanism of neuroprotection through DREAM silencing in HD does not apply to other NCS family members.

**Electronic supplementary material:**

The online version of this article (10.1186/s13041-018-0359-6) contains supplementary material, which is available to authorized users.

## Introduction

Huntington’s disease is a devastating, dominantly inherited neurodegenerative disorder caused by expansion of the number of CAG triplets in the first exon of the huntingtin (*htt*) gene. Expression of mutated Htt (mHtt) induces profound changes in calcium and protein homeostasis that lead ultimately to transcriptional deregulation and synaptic dysfunction [1]. Synaptic dysfunction in HD, and in other neurodegenerative diseases including Alzheimer’s and Parkinson’s diseases, is largely related to failed protein homeostasis because of a defective unfolded protein response (UPR) and accumulation of pathogenic protein aggregates at synapses [2–6]. Defective UPR in HD is associated with reduced ATF6 processing and a poor pro-adaptive UPR response in the striatum of patients and in HD mouse models [7, 8]. HD neuropathology involves the nearly total loss of motor, cognitive, and emotional control, which is associated with selective death of striatal medium spiny neurons as well as of cortical neurons, the majority of which project to the striatum [1].

DREAM, also known as calsenilin or KChIP3, is a NCS protein that regulates Ca^2+^ homeostasis and neuronal survival through transcriptional control of target genes and through protein-protein interactions [9–12]. Transcriptomic and ChIP-seq analyses showed that DREAM is a master-switch transcription factor that regulates the on/off status of specific Ca^2+^-dependent gene expression programs that control synaptic plasticity, learning, and memory [13, 14]. Long-term depression (LTD), a form of synaptic plasticity, contextual fear, and spatial memory, as well as behavioral anxiety are impaired in transgenic mice that overexpress a dominant active mutant of DREAM (daDREAM) [15]. DREAM-deficient mice show changes in fear conditioning tests [16, 17] and a slight increase in long-term potentiation (LTP) in the dentate gyrus of the hippocampal formation [18]. The mechanism involves postsynaptic modulation of NMDA (N-methyl-D-aspartic acid) receptors by DREAM through a Ca^2+^-dependent interaction with PSD-95 (post-synaptic density protein 95) [15], or by direct interaction with the NMDA-R1 subunit [19]. In addition, neuronal expression of daDREAM in daDREAM transgenic mice resulted in a complex phenotype that shows i) loss of recurrent inhibition and enhanced LTP in the dentate gyrus as well as impaired learning and memory [13], ii) changes in the expression of specific activity-dependent transcription factors in the hippocampus, including Npas4, Nr4a1 and c-Fos; in addition, these mice have iii) changes in the expression of genes related to the cytoskeleton such as Arc, formin 1 and gelsolin, which are responsible for specific changes in dendritic arborization and spine density in CA1 pyramidal neurons and granule cells of the dentate gyrus [14]. Together these changes recapitulate the role of DREAM in structural plasticity in the hippocampus.

DREAM expression is reduced in murine HD models and in HD patients compared to wild-type mice or healthy individuals [8]. In the R6/2 HD mouse model, decreased DREAM levels are detectable already a few weeks after birth, well before the onset of disease symptoms. Genetic experiments indicated that DREAM downregulation is part of an endogenous neuroprotective mechanism to counteract an equally early failure in ATF6 processing and UPR dysfunction [8]. Chronic administration of repaglinide, an oral hypoglycemic drug that interacts with DREAM, enhances ATF6 processing, which improves the UPR pro-survival function and reduces neuronal loss in the R6/2 mouse striatum [8]. The mechanism involves repaglinide disruption of the Ca^2+^-dependent DREAM-ATF6 interaction and nuclear accumulation of transcriptionally active ATF6. Improved UPR after chronic repaglinide administration results in delayed onset and slowed progression of motor disease symptoms [8].

Reduced DREAM mRNA levels are also observed in the hippocampus and the cerebral cortex in HD mouse models [8], and decreased nuclear ATF6 immunoreactivity was reported in cortical neurons from HD patients [7]. It is currently not known whether these observations are indicative of an equivalent neuroprotective mechanism by DREAM downregulation that prevents cognitive decline in HD. Here we show that blockade of DREAM activity by repaglinide or by induced DREAM haplodeficiency delayed onset of memory deficits in adult R6/1 mice. We also show that DREAM expression is reduced in the hippocampus of HD patients, while there is no change for other NCS proteins.

## Methods

### Mice and in vivo treatment

R6/1 mice were originally obtained from Jackson Laboratories. The colony was maintained by breeding male R6/1 with CBA × C57BL/6 mice to obtain heterozygous mutants and wild-type offspring. Genotype and CAG-repeat length were determined by PCR-based amplification using genomic DNA extracted from tail biopsies. Our R6/1 colony had an average repeat length close to 150 repeats, more than the110 repeats originally reported for this mutant transgenic line [20]. Only R6/1 mice with fewer than 170 CAG repeats were used in these experiments. Repaglinide (2 μg/ml) or vehicle (DMSO; 0.2 μl/ml) was administered chronically in drinking water shortly after weaning.

### Behavioral analysis

Experiments were performed in R6/1 mice and wild-type littermates of the indicated ages. Mice were initially housed five per cage in a temperature- (21 ± 1 °C) and humidity- (65 ± 10%) controlled room with a 12/12-h light/dark cycle (lights on from 08:00 to 20:00 h), with food and water ad libitum. All experiments took place during the light phase. All behavioral experiments were carried out in blind conditions for genotype and treatment.

The rotarod test was used to measure motor coordination and balance (Accelerating Model, Ugo Basile, Biological Research Apparatus). For basal rotarod performance, mice were tested on two consecutive days. On day 1 (training), each mouse was placed on the rotarod at a constant speed (4 r.p.m.) for a maximum of 60 s. The procedure was repeated three times with a rest period of 30 min between trials. On day 2 (experiment), mice received one training trial at constant speed (4 r.p.m.) for a maximum of 60 s, followed by three test trials with acceleration from 4 to 40 r.p.m. over a period of 60 s and the latency to fall off the rotarod within this period was recorded. Any mice remaining on the apparatus after 60 s were removed and their time scored as 60 s. Data from the three test trials were averaged for each animal and used for statistical analyses.

The novel object recognition test was performed as reported [21, 22]. In brief, mice were first individually habituated to the open-field for 50 min. The next day, they were submitted to a 10-min acquisition trial (first trial) during which they were placed in the open-field in the presence of object A. The time the animal took to explore object A (animal’s snout directed toward the object at a distance < 1 cm) was recorded. Two 10-min retention trials occurred 4 h later (second trial) and 24 h later (third trial). During the second and third trials, objects A and B (second trial) or C (third trial) were placed in the open-field, and the times the animal took to explore object A (tA) and the novel objects B or C (tN) were recorded. A discrimination index was defined as [(tN – tA)/(tA + tN)] × 100. Mice that explored less than five seconds during the initial ten minutes acquisition trial were excluded from the test. Similarly, mice exploring less than five seconds during a given retention trial (4 or 24 h) were excluded from that analysis.

### Western blot

Hippocampal whole cell extracts were prepared as described [14]. In brief, brain tissue was homogenized on ice in RIPA buffer (9806, Cell Signaling Technology) supplemented with protease inhibitor (cOmplete EDTA-free, Roche) and 1 mM PMSF (phenylmethanesulfonyl fluoride). Extracts were cleared by centrifugation (14,000 g, 20 min. 4 °C). Samples (20–30 μg) were analyzed by SDS-PAGE (sodium dodecyl sulfate-polyacrylamide gel electrophoresis) and immunoblot. PVDF (polyvinylidene difluoride) membranes were incubated (overnight, 4 °C) with specific antibodies to DREAM (Ab731, [23]), ATF6α (A303–719, Bethyl), hippocalcin (G-8, Santa Cruz), VILIP-1 (C-term, Abgent) or to NCS-1 (C-15, Santa Cruz). Equal protein loading was measured by Coomassie staining of the membrane after immunoblotting (Additional file 1). Secondary antibodies used were HRP (horseradish peroxidase)-conjugated donkey anti-rabbit, −mouse or -goat IgG antibody (Jackson) and detection was with ECL Select (GE Healthcare). Lane and band intensity were quantified with ImageLab software (BioRad).

### Statistical analyses

All data values are shown as mean ± SEM. Differences were considered significant at *P* < 0.05. When possible, two-way ANOVA was used to analyze statistical differences among groups. In the case of unequal or small sample size or non-Gaussian distribution, comparisons between groups were analyzed using the nonparametric ANOVA, Kruskal-Wallis test with Dunn’s multiple comparisons between groups. Two-group comparison was performed with unpaired 2-tailed Student’s t test. Animal experiments were randomized. Sample size was not predetermined by statistical method. Prism GraphPad Software 6.0 was used to plot graphs and for statistical analysis.

## Results

### Chronic repaglinide administration delays onset of memory impairment in R6/1 mice

**Fig. 1:**
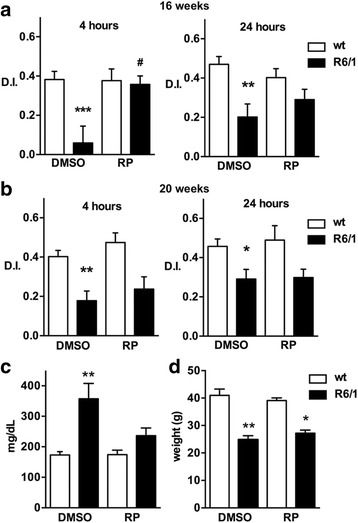
Repaglinide delays the onset of cognitive impairment in R6/1 mice. Memory was assessed in 16- (**a**) and 20-weeks (**b**) wt or R6/1 old mice using the novel object recognition test. Mice received repaglinide (RP) or vehicle (DMSO) in drinking water from shortly after weaning. The discrimination index (D.I.) reflects the ability to recognize novelty 4 or 24 h after first exposure to the object. The number of mice included in the novel object recognition test: wt-DMSO (26–31), wt-RP (14–21), R6/1-DMSO (18–37), R6/1-RP (12–20). **c** Circulating glucose levels in 20-week-old mice of the indicated genotype and treatment. After overnight fasting, mice had access to food pellets for 1 h before testing. The number of mice used for glucose determination: wt-DMSO (10), wt-RP (15), R6/1-DMSO (10), R6/1-RP (20). **d** Body weight of 20-week-old male mice of the indicated genotype and treatment. The number of mice used for body weight determination: wt-DMSO (9), wt-RP (6), R6/1-DMSO (6), R6/1-RP (6). Data are shown as mean ± SEM. Non-parametric ANOVA, Kruskal-Wallis test (*P* values: a) 0.0014 and 0.006 for 4 and 24 h, respectively; b) 0.0003 and 0.0118 for 4 and 24 h, respectively; c) 0.0017; d) 0.0009) with Dunn’s multiple comparisons between selected groups was used, *** *p* <0.005, ** *p* <0.01, * *p* <0.05 vs wt-DMSO, # *p* <0.05, R6/1-RP vs R6/1-DMSO

Using the novel object recognition test, we found that short- and long-term memory were significantly impaired at early stages of the Huntington’s pathology in R6/1 mice. These data confirmed previous reports [24–26]. The cognitive decline was already noticeable at 16 weeks after birth (Fig. 1a). Chronic repaglinide administration (2 μg/ml; ad lib in drinking water), which was begun shortly after weaning, partially prevented this decline and restored discrimination ability at 4 h after the test (short-term memory). Repaglinide nonetheless did not improve cognition decline when tested 24 h later (long-term memory) (Fig. 1a). Impaired short- and long-term memory were also observed in 20-week-old R6/1 mice, although chronic repaglinide administration had no effect on cognition loss at this disease stage (Fig. 1b); repaglinide nonetheless effectively reduced the post-prandial increase in circulating glucose levels in 20-week-old R6/1 mice (Fig. 1c). As reported in R6/2 mice [8], extended repaglinide administration in R6/1 or wild-type littermates produced no obvious adverse effects and had no effect on progressive body weight loss in transgenic R6/1 mice (Fig. 1d).

**Fig. 2:**
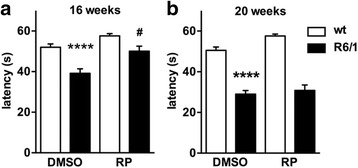
Latency to fall in the rotarod test. Motor coordination was assessed in 16- (**a**) and 20-weeks (**b**) wt or R6/1 old mice using the rotarod test. Mice of indicated genotypes and ages were exposed chronically to vehicle or repaglinide. The number of mice used: wt-DMSO (35), wt-RP (20), R6/1-DMSO (30), R6/1-RP (24). Data are shown as mean ± SEM. Nonparametric ANOVA, Kruskal-Wallis test (*P* values for both panels < 0.0001) with Dunn’s multiple comparisons between selected groups was used. **** *p* < 0.0001 vs wt-DMSO; # *p* < 0.05 R6/1-RP vs R6/1-DMSO

Parallel assessment of R6/1 mice in the rotarod test showed impaired motor coordination at 16 weeks, which became more pronounced by 20 weeks after birth (Fig. 2), as reported [24–26]. Chronic repaglinide administration blocked motor dysfunction at 16 weeks, but had no effect in 20-week-old R6/1 mice (Fig. 2). The transient effect of repaglinide in R6/1 mice is similar to its lack of effect on motor coordination in R6/2 mice at more advanced stages of the disease [8].

**Fig. 3:**
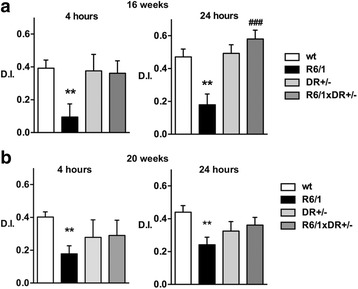
Induced DREAM haplodeficiency delays onset of cognitive impairment in R6/1 mice. Memory was assessed in 16- (**a**) and 20-weeks (**b**) old mice using the novel object recognition test. The number of mice used: wt (21), R6/1 (20–23), DREAM^+/−^ (9–12), R6/1× DREAM^+/−^ (9–12). DREAM haplodeficiency is indicated as D^+/−^. Data are shown as mean ± SEM. Non-parametric ANOVA, Kruskal-Wallis test (*P* values: a) 0.0262 and 0.0002 for 4 and 24 h, respectively; b) 0.0122 and 0.0187 for 4 and 24 h, respectively) with Dunn’s multiple comparisons between selected groups were used. *** *p* <0.005, ** *p* <0.01, R6/1 vs wt, ### *p* <0.005, R6/1xDR^+/−^ vs R6/1xD^+/+^

Ablation of one copy of DREAM in double transgenic R6/1xDREAM^+/−^ mice significantly improved the discrimination index at 4 and 24 h after exposure to the novel objects in 16-week-old mice, whereas the effect was not significant in 20-week-old mice (Fig. 3). These results parallel the delay of the onset of motor symptoms in R6/2 mice after induced DREAM haplodeficiency [8].

### Repaglinide administration normalizes ATF6 processing in the R6/1 mouse hippocampus

Reduced DREAM mRNA expression and protein levels in the brain of R6/1 and R6/2 mice is a neuroprotective response, and further inhibition of DREAM activity is associated with improved motor coordination in the HD mice treated with repaglinide [8]. To test whether a similar mechanism is involved in the partial protection from cognitive decline in repaglinide-treated HD mice, we analyzed the effect of repaglinide administration on ATF6 processing in R6/1 hippocampus, a brain area involved in learning and memory [27].

**Fig. 4:**
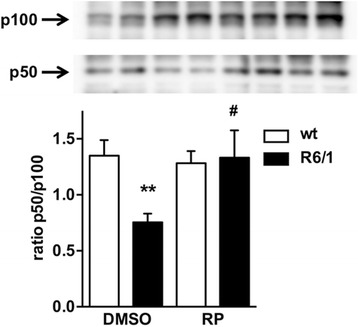
Repaglinide activates ATF6 processing in mouse hippocampal neurons. Western blot analysis of whole cell extracts from hippocampus of wild-type and R6/1 mice receiving DMSO or repaglinide. A representative blot is shown. Bands corresponding to full-length ATF6 (p100) and processed N-terminal ATF6 (p50) are shown. The normalized p50/p100 ratio based on quantification of 5 experiments is shown. Data are shown as mean ± SEM. Nonparametric ANOVA, Kruskal-Wallis test (*P* = 0.0024) with Dunn’s multiple comparisons between selected groups were used. ** *p* < 0.01, R6/1 vs wt-DMSO, # *p* < 0.05, R6/1-RP vs R6/1-DMSO

ATF6 processing, measured as the ratio between the processed (p50) and the full-length (p100) forms of the protein, was markedly reduced in the R6/1 mouse hippocampus compared to wild-type littermates (Fig. 4). Repaglinide normalized ATF6 processing in the R6/1 mouse hippocampus without changing ATF6 processing in wild-type littermates (Fig. 4). Recovery of ATF6 processing in the R6/1 hippocampus thus correlates with delayed cognition impairment after repaglinide administration.

### Differential changes in NCS protein expression in the hippocampus of HD patients

Repaglinide binds to DREAM and to other members of the NCS superfamily [8, 28], and changes in expression of some NCS proteins have been described in HD patients [29]. We therefore analyzed whether expression of three NCS proteins other than DREAM is also reduced in hippocampus from HD patients, and might be targeted by repaglinide.

**Fig. 5:**
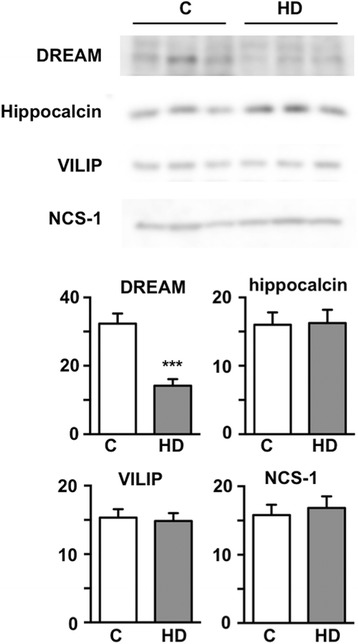
DREAM levels are reduced in hippocampus from HD patients. Western blot analysis of the indicated NCS proteins in hippocampus from controls (C) (*n* = 5) and HD patients (*n* = 7). Representative blots are shown. The experiment was repeated three times. Case information for human samples used in the study is shown in Additional file 2. Quantified intensity ratio vs loading control (Coomassie staining) is shown. Unpaired two-tailed Student’s *t*-test. *** *p* < 0.005, HD vs C

As shown in the striatum from HD patients [8], DREAM protein levels are notably reduced in hippocampal samples from HD patients compared to control samples (Fig. 5). Neuronal calcium sensor-1 (NCS-1), visinin-like protein (VILIP-1) and hippocalcin levels were nonetheless similar in the same control and HD hippocampus samples (Fig. 5). These results further contribute to the idea that reduced DREAM levels in the hippocampus of HD patients may have a neuroprotective effect to limit and/or slow down cognitive decline in HD.

## Discussion

Early synaptic dysfunction and progressive accumulation of pathogenic protein aggregates (e.g., huntingtin inclusions, amyloid plaques, and neurofibrillary tangles) lead to gradual, inescapable cognitive impairment and neuronal death in HD and Alzheimer’s disease (AD). In the case of HD, early symptoms of synaptic dysfunction in the cortico-striatal pathway include changes in NMDA receptor signaling, reduced response to external stimuli (e.g., impaired induction of activity-dependent genes), progressive loss of synaptic contacts (e.g., post-synaptic dendritic spines in excitatory synapses), and gradual degeneration of medium-sized striatal spiny neurons [30]. Reduction in hippocampal volume is reported in HD patients [31, 32], which correlates with altered spatial short-term and recognition memories in these patients [33, 34]. Cognitive decline precedes motor manifestations, both in patients and in HD mouse models [35–37].

The NCS superfamily is encoded by 14 genes in mammals and, through alternative splicing, encompasses more than 40 isoforms [38, 39]. Guided by multiple Ca^2+^-dependent and -independent protein-protein interactions, and by a specific Ca^2+^-dependent interaction with DNA in DREAM/KChIP subfamily members [9, 40], NCS proteins participate in numerous physiological functions [38, 39]. In addition, changes in the expression of or mutations in NCS proteins are associated with several neurological disorders. NCS-1 is upregulated in patients with schizophrenia or bipolar disorder [41] as well as in the substantia nigra from Parkinson’s disease patients [42], and a mutation in NCS-1 was found in a case of autistic spectrum disorder [43, 44]. Expression of neurocalcin [45] and VILIP-1 [46] were reduced in AD brains compared with age-matched brain samples. Increased VILIP-1 levels in cerebrospinal fluid were proposed as diagnostic and prognostic biomarkers of neuroinflammation and cognitive decline in patients with AD, dementia with Lewy bodies, or frontotemporal lobar degeneration [47–53]. Finally, decreased DREAM mRNA and protein levels [8] and hippocalcin mRNA expression [29] were found in striatum from Huntington’s disease patients. This reduction in hippocalcin mRNA nonetheless did not correlate with striatal vulnerability, and the study did not analyze hippocalcin protein levels. Our results show that, of the NCS proteins tested, only DREAM levels were decreased in hippocampal samples from HD patients. These results further extend DREAM involvement in HD, and suggest that NCS-1, hippocalcin, and VILIP-1 are not implicated functionally in this neurodegenerative pathology.

Early downregulation of DREAM expression in the HD mouse striatum is associated with an ATF6-mediated neuroprotective mechanism that delays onset and slows progression of the motor symptoms of the disease [8]. Here we show that DREAM and transcriptionally active ATF6 are also reduced in the hippocampus and that chronic administration of repaglinide improves ATF6 processing and delays memory impairment in adult R6/1 mice. Removal of one copy of DREAM in R6/1xDREAM^+/−^ double transgenic mice similarly delayed onset of cognitive loss, as assayed in the novel object recognition test. As shown for improved motor coordination in R6/2 mice [8], pharmacologically or genetically induced reduction of DREAM activity in R6/1 mice also has a transient effect at the hippocampal level, as cognitive loss was delayed but not prevented in advanced disease stages. The molecular basis for this finding is presently unknown, although it might be related to the activation of additional signaling pathways that lead to irreversible neuronal death and loss of cognitive function. Reduction of mHtt mRNA levels using sustained, neuron-specific expression of synthetic zinc finger constructs that target the CAG repeats in the *mHtt* gene also provided only transient protection for neuronal loss in R6/1 mice [54].

Repaglinide binding to NCS proteins was first reported in bovine brain and retinal extracts, which showed Ca^2+^-dependent binding respectively to neurocalcin and VILIP-1, or to recoverin [28]. Repaglinide also binds to members of the DREAM/KChIP subfamily [8], which indicates that repaglinide binding is a characteristic of all proteins of the NCS superfamily. After binding, repaglinide interferes with the biological activity of the Ca^2+^ sensor, that is, it blocks recoverin-mediated inhibition of rhodopsin kinase activity or DREAM-induced suppression of ATF6 processing [8, 28].

Repaglinide was developed as a potent insulinotropic agent for treatment of type-2 diabetes [55]. The mechanism involves the blockade of ATP-dependent potassium channels, which induces insulin release. Like glibenclamide, another insulinotropic molecule, nanomolar concentrations of repaglinide block these channels. Repaglinide binds to DREAM and blocks activation of Kv4 potassium channels, also at nanomolar concentrations, whereas glibenclamide is inactive [8]. The interaction of repaglinide with NCS proteins is specific for this group, and does not occur with other Ca^2+^-binding proteins, including calmodulin or proteins of the S-100 superfamily [28].

Our results suggest that DREAM inhibition has a role in delaying cognitive decline in HD mice through a mechanism related to ATF6 processing. This neuroprotection through DREAM silencing in HD is specific, and does not apply to other NCS family members. Ongoing studies that include molecular docking and structure-activity relationship analysis will help to better understand this interaction and, ideally, to define new ligands with improved selectivity for the different NCS subfamilies.

## Additional files

Additional file 1: Coomassie staining of total protein was used to confirm equivalent protein loading, shown for representative blots in (a) Fig. 4 and (b) Fig. 5. (TIFF 196 kb)

Additional file 2: Case information for human samples used in this study. (DOCX 43 kb)

## Abbreviations

AD: Alzheimer’s disease
ATF6: Activating transcription factor 6
DREAM: Downstream antagonist modulator
HD: Huntington’s disease
KChIP: Potassium channel interactive protein
LTD: Long-term depression
LTP: Long-term potentiation
mHtt: Mutated huntingtin protein
NCS: Neuronal calcium sensor
NCS-1: Neuronal calcium sensor 1
UPR: Unfolded protein response
VILIP-1: Visinin-like protein 1

## References

[1] Saudou F, Humbert S (2016). The biology of huntingtin. Neuron.

[2] Lee J, Ozcan U (2014). Unfolded protein response signaling and metabolic diseases. J Biol Chem.

[3] Halliday M, Mallucci GR (2015). Modulating the unfolded protein response to prevent neurodegeneration and enhance memory. Neuropathol Appl Neurobiol.

[4] Hetz C, Mollereau B (2014). Disturbance of endoplasmic reticulum proteostasis in neurodegenerative diseases. Nat Rev Neurosci.

[5] Sprenkle NT, Sims SG, Sanchez CL, Meares GP (2017). Endoplasmic reticulum stress and inflammation in the central nervous system. Mol Neurodegener.

[6] Herms J, Dorostkar MM (2016). Dendritic spine pathology in neurodegenerative diseases. Annu Rev Pathol.

[7] Fernandez-Fernandez MR, Ferrer I, Lucas JJ (2011). Impaired ATF6alpha processing, decreased Rheb and neuronal cell cycle re-entry in Huntington's disease. Neurobiol Dis.

[8] Naranjo JRZH, Villar D, González P, Dopazo XM, Morón J, Higueras E, Oliveros JC, Arrabal MD, Prieto A, Cercós P, González T, De la Cruz A, Casado-Vela J, Rábano A, Valenzuela C, Gutierrez-Rodriguez M, Li JY, Mellström B (2016). Activating transcription factor 6 de-repression mediates neuroprotection in Huntington's disease. J Clin Invest.

[9] Carrion AM, Link WA, Ledo F, Mellstrom B, Naranjo JR (1999). DREAM is a Ca2+−regulated transcriptional repressor. Nature.

[10] Buxbaum JD, Choi EK, Luo Y, Lilliehook C, Crowley AC, Merriam DE, Wasco W (1998). Calsenilin: a calcium-binding protein that interacts with the presenilins and regulates the levels of a presenilin fragment. Nat Med.

[11] An WF, Bowlby MR, Betty M, Cao J, Ling HP, Mendoza G, Hinson JW, Mattsson KI, Strassle BW, Trimmer JS (2000). Modulation of A-type potassium channels by a family of calcium sensors. Nature.

[12] Mellstrom B, Savignac M, Gomez-Villafuertes R, Naranjo JR (2008). Ca2+−operated transcriptional networks: molecular mechanisms and in vivo models. Physiol Rev.

[13] Mellstrom B, Sahun I, Ruiz-Nuno A, Murtra P, Gomez-Villafuertes R, Savignac M, Oliveros JC, Gonzalez P, Kastanauskaite A, Knafo S (2014). DREAM controls the on/off switch of specific activity-dependent transcription pathways. Mol Cell Biol.

[14] Mellström BKA, Knafo S, Gonzalez P, Dopazo XM, Ruiz-Nuño A, Jefferys J, Zhuo M, Bliss TVP, Naranjo JR, De Felipe J, Unbalanced DREAM (2016). Activity modifies hippocampal connectivity and cognition. Mol Brain.

[15] Wu LJ, Mellstrom B, Wang H, Ren M, Domingo S, Kim SS, Li XY, Chen T, Naranjo JR, Zhuo M (2010). DREAM (downstream regulatory element antagonist modulator) contributes to synaptic depression and contextual fear memory. Mol Brain.

[16] Lilliehook C, Bozdagi O, Yao J, Gomez-Ramirez M, Zaidi NF, Wasco W, Gandy S, Santucci AC, Haroutunian V, Huntley GW (2003). Altered Abeta formation and long-term potentiation in a calsenilin knock-out. J Neurosci.

[17] Alexander JC, McDermott CM, Tunur T, Rands V, Stelly C, Karhson D, Bowlby MR, An WF, Sweatt JD, Schrader LA (2009). The role of calsenilin/DREAM/KChIP3 in contextual fear conditioning. Learn Mem.

[18] Cheng HY, Pitcher GM, Laviolette SR, Whishaw IQ, Tong KI, Kockeritz LK, Wada T, Joza NA, Crackower M, Goncalves J (2002). DREAM is a critical transcriptional repressor for pain modulation. Cell.

[19] Zhang Y, Su P, Liang P, Liu T, Liu X, Liu XY, Zhang B, Han T, Zhu YB, Yin DM (2010). The DREAM protein negatively regulates the NMDA receptor through interaction with the NR1 subunit. J Neurosci.

[20] Mangiarini L, Sathasivam K, Seller M, Cozens B, Harper A, Hetherington C, Lawton M, Trottier Y, Lehrach H, Davies SW (1996). Exon 1 of the HD gene with an expanded CAG repeat is sufficient to cause a progressive neurological phenotype in transgenic mice. Cell.

[21] Tan VTY, Mockett BG, Ohline SM, Parfitt KD, Wicky HE, Peppercorn K, Schoderboeck L, Yahaya MFB, Tate WP, Hughes SM (2018). Lentivirus-mediated expression of human secreted amyloid precursor protein-alpha prevents development of memory and plasticity deficits in a mouse model of Alzheimer's disease. Mol Brain.

[22] Cui L, Sun W, Yu M, Li N, Guo L, Gu H, Zhou Y (2016). Disrupted-in-schizophrenia1 (DISC1) L100P mutation alters synaptic transmission and plasticity in the hippocampus and causes recognition memory deficits. Mol Brain.

[23] Savignac M, Pintado B, Gutierrez-Adan A, Palczewska M, Mellstrom B, Naranjo JR (2005). Transcriptional repressor DREAM regulates T-lymphocyte proliferation and cytokine gene expression. EMBO J.

[24] van der Borght K, Brundin P (2007). Reduced expression of PSA-NCAM in the hippocampus and piriform cortex of the R6/1 and R6/2 mouse models of Huntington's disease. Exp Neurol.

[25] Li W, Silva HB, Real J, Wang YM, Rial D, Li P, Payen MP, Zhou Y, Muller CE, Tome AR (2015). Inactivation of adenosine A2A receptors reverses working memory deficits at early stages of Huntington's disease models. Neurobiol Dis.

[26] Tyebji S, Saavedra A, Canas PM, Pliassova A, Delgado-Garcia JM, Alberch J, Cunha RA, Gruart A, Perez-Navarro E (2015). Hyperactivation of D1 and A2A receptors contributes to cognitive dysfunction in Huntington's disease. Neurobiol Dis.

[27] Eichenbaum H (2001). The hippocampus and declarative memory: cognitive mechanisms and neural codes. Behav Brain Res.

[28] Okada M, Takezawa D, Tachibanaki S, Kawamura S, Tokumitsu H, Kobayashi R (2003). Neuronal calcium sensor proteins are direct targets of the insulinotropic agent repaglinide. Biochem J.

[29] Rudinskiy N, Kaneko YA, Beesen AA, Gokce O, Regulier E, Deglon N, Luthi-Carter R (2009). Diminished hippocalcin expression in Huntington's disease brain does not account for increased striatal neuron vulnerability as assessed in primary neurons. J Neurochem.

[30] Bunner KD, Rebec GV (2016). Corticostriatal dysfunction in Huntington's disease: the basics. Front Hum Neurosci.

[31] Rosas HD, Koroshetz WJ, Chen YI, Skeuse C, Vangel M, Cudkowicz ME, Caplan K, Marek K, Seidman LJ, Makris N (2003). Evidence for more widespread cerebral pathology in early HD: an MRI-based morphometric analysis. Neurology.

[32] Ille R, Schafer A, Scharmuller W, Enzinger C, Schoggl H, Kapfhammer HP, Schienle A (2011). Emotion recognition and experience in Huntington disease: a voxel-based morphometry study. J Psychiatry Neurosci.

[33] Lemiere J, Decruyenaere M, Evers-Kiebooms G, Vandenbussche E, Dom R (2004). Cognitive changes in patients with Huntington's disease (HD) and asymptomatic carriers of the HD mutation--a longitudinal follow-up study. J Neurol.

[34] Montoya A, Pelletier M, Menear M, Duplessis E, Richer F, Lepage M (2006). Episodic memory impairment in Huntington's disease: a meta-analysis. Neuropsychologia.

[35] Paulsen JS (2011). Cognitive impairment in Huntington disease: diagnosis and treatment. Curr Neurol Neurosci Rep.

[36] Stout JC, Paulsen JS, Queller S, Solomon AC, Whitlock KB, Campbell JC, Carlozzi N, Duff K, Beglinger LJ, Langbehn DR (2011). Neurocognitive signs in prodromal Huntington disease. Neuropsychology.

[37] Puigdellivol M, Saavedra A, Perez-Navarro E (2016). Cognitive dysfunction in Huntington's disease: mechanisms and therapeutic strategies beyond BDNF. Brain Pathol.

[38] Burgoyne RD, Haynes LP (2012). Understanding the physiological roles of the neuronal calcium sensor proteins. Mol Brain.

[39] Burgoyne RD, Haynes LP (2015). Sense and specificity in neuronal calcium signalling. Biochim Biophys Acta.

[40] Link WA, Ledo F, Torres B, Palczewska M, Madsen TM, Savignac M, Albar JP, Mellstrom B, Naranjo JR (2004). Day-night changes in downstream regulatory element antagonist modulator/potassium channel interacting protein activity contribute to circadian gene expression in pineal gland. J Neurosci.

[41] Koh PO, Undie AS, Kabbani N, Levenson R, Goldman-Rakic PS, Lidow MS (2003). Up-regulation of neuronal calcium sensor-1 (NCS-1) in the prefrontal cortex of schizophrenic and bipolar patients. Proc Natl Acad Sci U S A.

[42] Dragicevic E, Poetschke C, Duda J, Schlaudraff F, Lammel S, Schiemann J, Fauler M, Hetzel A, Watanabe M, Lujan R (2014). Cav1.3 channels control D2-autoreceptor responses via NCS-1 in substantia nigra dopamine neurons. Brain.

[43] Piton A, Michaud JL, Peng H, Aradhya S, Gauthier J, Mottron L, Champagne N, Lafreniere RG, Hamdan FF, team SD (2008). Mutations in the calcium-related gene IL1RAPL1 are associated with autism. Hum Mol Genet.

[44] Handley MT, Lian LY, Haynes LP, Burgoyne RD (2010). Structural and functional deficits in a neuronal calcium sensor-1 mutant identified in a case of autistic spectrum disorder. PLoS One.

[45] Shimohama S, Chachin M, Taniguchi T, Hidaka H, Kimura J (1996). Changes of neurocalcin, a calcium-binding protein, in the brain of patients with Alzheimer's disease. Brain Res.

[46] Braunewell K, Riederer P, Spilker C, Gundelfinger ED, Bogerts B, Bernstein HG (2001). Abnormal localization of two neuronal calcium sensor proteins, visinin-like proteins (vilips)-1 and −3, in neocortical brain areas of Alzheimer disease patients. Dement Geriatr Cogn Disord.

[47] Tarawneh R, D'Angelo G, Macy E, Xiong C, Carter D, Cairns NJ, Fagan AM, Head D, Mintun MA, Ladenson JH (2011). Visinin-like protein-1: diagnostic and prognostic biomarker in Alzheimer disease. Ann Neurol.

[48] Tarawneh R, Lee JM, Ladenson JH, Morris JC, Holtzman DM (2012). CSF VILIP-1 predicts rates of cognitive decline in early Alzheimer disease. Neurology.

[49] Luo X, Hou L, Shi H, Zhong X, Zhang Y, Zheng D, Tan Y, Hu G, Mu N, Chan J (2013). CSF levels of the neuronal injury biomarker visinin-like protein-1 in Alzheimer's disease and dementia with Lewy bodies. J Neurochem.

[50] Mroczko B, Groblewska M, Zboch M, Muszynski P, Zajkowska A, Borawska R, Szmitkowski M, Kornhuber J, Lewczuk P (2015). Evaluation of visinin-like protein 1 concentrations in the cerebrospinal fluid of patients with mild cognitive impairment as a dynamic biomarker of Alzheimer's disease. J Alzheimers Dis.

[51] Kirkwood CM, MacDonald ML, Schempf TA, Vatsavayi AV, Ikonomovic MD, Koppel JL, Ding Y, Sun M, Kofler JK, Lopez OL (2016). Altered levels of Visinin-like protein 1 correspond to regional neuronal loss in Alzheimer disease and frontotemporal lobar degeneration. J Neuropathol Exp Neurol.

[52] Cicognola C, Chiasserini D, Eusebi P, Andreasson U, Vanderstichele H, Zetterberg H, Parnetti L, Blennow K (2016). No diurnal variation of classical and candidate biomarkers of Alzheimer's disease in CSF. Mol Neurodegener.

[53] Muszynski P, Kulczynska-Przybik A, Borawska R, Litman-Zawadzka A, Slowik A, Klimkowicz-Mrowiec A, Pera J, Dziedzic T, Mroczko B (2017). The relationship between markers of inflammation and degeneration in the central nervous system and the blood-brain barrier impairment in Alzheimer's disease. J Alzheimers Dis.

[54] Agustin-Pavon C, Mielcarek M, Garriga-Canut M, Isalan M (2016). Deimmunization for gene therapy: host matching of synthetic zinc finger constructs enables long-term mutant huntingtin repression in mice. Mol Neurodegener.

[55] Malaisse WJ (1995). Stimulation of insulin release by non-sulfonylurea hypoglycemic agents: the meglitinide family. Horm Metab Res.

