# Supplementary figures and images for: Inhibition of DREAM-ATF6 interaction delays onset of cognition deficit in a mouse model of Huntington’s disease
Source: Mol Brain. 2018 Mar 9;11:13. doi: 10.1186/s13041-018-0359-6 (PMC5845147; doi:10.1186/s13041-018-0359-6)

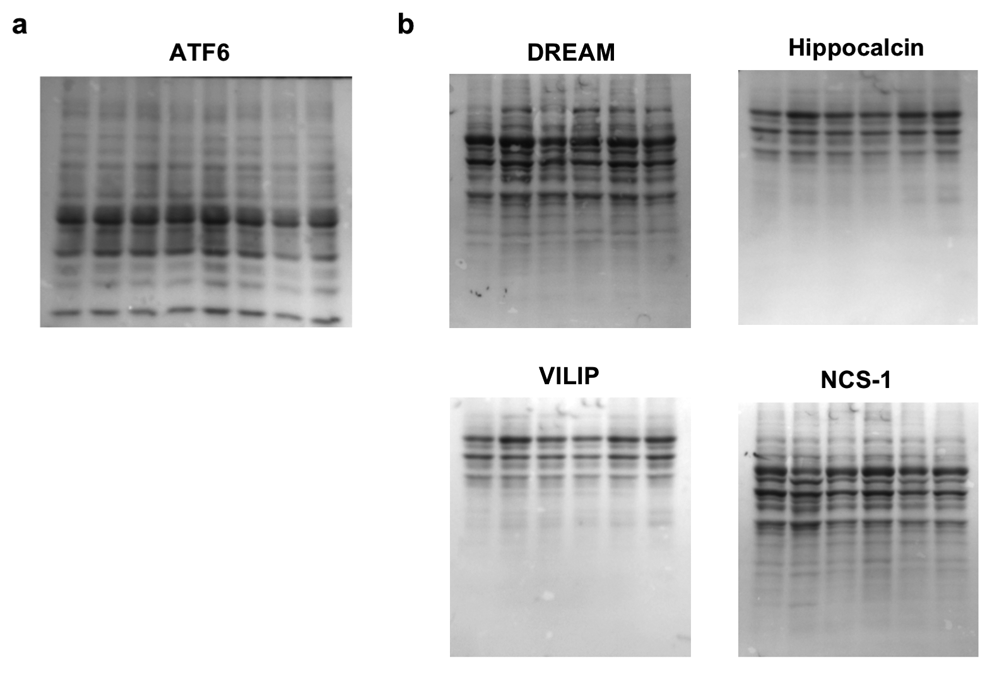

Supplement: Supplementary file 1 — Coomassie staining of total protein was used to confirm equivalent protein loading, shown for representative blots in (a) Fig. 4 and (b) Fig. 5. (TIFF 196 kb) [file 13041_2018_359_MOESM1_ESM.tif]
