# Supplementary material for: Inhibition of DREAM-ATF6 interaction delays onset of cognition deficit in a mouse model of Huntington’s disease
Source: Mol Brain. 2018 Mar 9;11:13. doi: 10.1186/s13041-018-0359-6 (PMC5845147; doi:10.1186/s13041-018-0359-6)
Supplement: Supplementary file 2 — Case information for human samples used in this study. (DOCX 43 kb) [file 13041_2018_359_MOESM2_ESM.docx]

**Additional File 2.** Case information for human samples used in this study?

| code | sex | age | phenotype |
| --- | --- | --- | --- |
| BCPA0364 | M | 43 | C |
| BCPA0537 | F | 74 | C |
| BCPA0587 | F | 83 | C |
| BCPA0662 | F | 58 | C |
| BCPA0720 | M | 61 | C |
| BCPA0324 | M | 56 | HD |
| BCPA0412 | M | 68 | HD |
| BCPA0703 | F | 56 | HD |
| BCPA0704 | M | 58 | HD |
| BCPA0711 | F | 64 | HD |
| BCPA0719 | M | 47 | HD |
| BCPA0733 | F | 68 | HD |
